# ANDORRA AS A LIVING LAB? THE INSCI EXAMPLE

**DOI:** 10.2340/jrm.v58.44222

**Published:** 2026-03-23

**Authors:** Mercè AVELLANET, Gerold STUCKI, Esther PAGES, Anna BOADA-PLADELLORENS, Christian GRILLO, Juli MINOVES-TRIQUEL, Jerome BICKENBACH

**Affiliations:** 1 Rehabilitation Department Hospital Nostra Sara de Meritxell, Andorra; 2 Research Group on Health Sciences, Universitat d’Andorra, Andorra, Spain; 3 Faculty of Health Sciences and Medicine, University of Lucerne, Lucerne; 4 Center for Rehabilitation in Global Health Systems, WHO Collaborating Center, University of Lucerne, Lucerne; 5 Swiss Paraplegic Research, Nottwil, Switzerland; 6 Rector, Universitat d’Andorra, Andorra, Spain

**Keywords:** research, health information systems, spinal cord injuries

## Abstract

**Objective:**

In light of the persistent concern identified by the World Health Organization (WHO) that small population countries tend to be ignored in international health research, preventing them from developing research capacity, this paper describes the participation of Andorra in an international spinal cord injury survey (InSCI) and resulting benefits.

**Methods:**

Descriptive analysis of Andorra’s health research situation and participation in InSCI.

**Results:**

Andorra has successfully participated in an international survey improving health research capacity and governmental support.

**Conclusion:**

In line with WHO recommendations to improve small country health research capacity, and specifically to improve their health information collection capacity, the described participation of Andorra in an international health survey demonstrates how this capacity can be improved without sacrificing methodological restrictions.

## LAY ABSTRACT

Because of their low population, small countries of the world tend not to participate in international health research, preventing them from developing their research capacity and gaining access to valuable comparative health data. The recent example of Andorra and its participation in an international spinal cord injury survey (InSCI) is described here to make the argument that the needs of these small countries to improve their health research capacity, and to collect valuable population data, can be addressed through active participation in international studies without diluting the methodological rigour of these studies.

## THE RESEARCH CHALLENGE OF SMALL COUNTRIES

The First High-level Meeting of Small Countries, under the auspices of the World Health Organization European Region (WHO-EURO), was held in San Marino on 3–4 July 2014, and focused on strengthening health information systems (HIS) and implementing the Health 2020 vision within Europe. The Small Countries Initiative had begun the year before as a network of European countries with a population of less than 2 million (Andorra, Cyprus, Estonia, Iceland, Latvia, Luxembourg, Malta, Monaco, Montenegro, North Macedonia, San Marino, and Slovenia) (1). The report was the result of the Small Countries initiative in 2013, which was intended as a “laboratory for innovation and solutions for health-related needs in small countries” (2). The Initiative was, and continues to be, coordinated by WHO-EURO. In 2022, a Roadmap towards better health in small countries in the WHO European Region, 2022–2025 was released (3). The Roadmap was strongly influenced by the lessons learned during the COVID-19 pandemic, which hit small countries hard, and amplified pre-existing challenges of dependency on larger neighbouring countries for access to medicines and healthcare workers. The Roadmap, as a result, focused on emergency preparedness.

The original motivation for the Small Countries Initiative, however, was to address a common research and data challenge: although small countries have the advantage of closer cooperation between health systems stakeholders – which facilitates implementation – their small population size means fewer qualified health researchers. Specialists often can only be trained outside of the country concerned, and after training they may be tempted to emigrate because of more opportunities in larger countries. Physical resources for data collection and storage may also be limited. Ultimately this creates a problem of lack of health research data. Studies indicate that for established health indicators the extent of missing data is considerable higher in small countries than in larger countries (4, 5). Missing data means fewer opportunities for evaluating the performance of the health system, and a constrained capacity to forecast future needs and plan accordingly. So too the capacity for international comparison using common European health indicators is compromised.

The common concern regarding the state of health information in small countries led in 2016 to WHO-EURO establishing a Small Countries Health Information Network (SCHIN) to enable small countries to work together in order to strengthen and improve their health information systems and research capacity (6). The Network reported that 2 kinds of challenges confront small countries: lack of technical and administrative capacity to handle health information, and a lack of legal and national strategies relevant for HIS improvement.

On the technical side, small countries may not have the specialized expertise of complex data analyses, leading to underutilization of collected data. Because of their small population, these countries may find it difficult to acquire minimal samples for surveys, or to avoid survey fatigue from multiple requests from the same small cohort of respondents. The small numbers may lead to data fluctuations and wide confidence intervals, undermining confidence in the quality of the data submitted. Small countries may also lack a tradition of health information collection as well as the legal and administrative framework for sustaining one. The required bureaucratic procedures, including to secure data privacy, may not be in place, and the country may lack any national strategic policies to support academics, researchers, and other stakeholders in collaborating to produce, analyse, and utilize health information.

Networking between small countries to find common solutions to similar challenges and also international collaborations are, as the WHO-EURO has made clear, essential tools for increasing the capacity of small countries to collect, analyse, and utilize health information, as a precondition to enhancing HIS capacity and strengthening health systems. On the other hand, there is much to learn for larger countries facing new financial and other constraints from how small countries have had to deal with their health information and research challenges.

The objective of this paper is to describe an example of a small country – in this case Andorra, which has been part of the WHO-EURO small country network from the beginning – and its participation in the International Spinal Cord Injury community survey (InSCI). In order to successfully participate in this large, 40-country survey, Andorra has had to become a kind of living lab for addressing small country health information challenges. Lessons learned here will apply not only to other small countries, but to larger ones as well.

### Andorra

Andorra is a small state located in the Pyrenees mountains between France and Spain. It is one of the smallest countries in Europe with a population of around 88,000 people and is governed as a parliamentary co-principality. In 2024, Andorra’s GDP per capita was estimated by the Andorran Government at US$42,853, placing it among the higher-income nations globally (estadistica.ad). Andorra’s economy is based on tourism, finance, and retail, and the country is known for its high standard of living and safety. Andorra’s healthcare system is also highly regarded, consistently ranking at or near the top in global assessments. Based on data from the 2018 Global Burden of Disease Study, Andorra scored 95 out of 100 on the Healthcare Access and Quality Index (HAQ) (7). The country has a single national hospital with 150 beds, including 12 ICU beds, and 12 primary care centres, but no tertiary care centres. There are approximately 500 physicians practising in Andorra and although most specialties are covered, highly specific expertise, such as cardiac and thoracic surgery, are not. As for high-impact but low prevalence conditions such as spinal cord injury, most patients are initially referred to facilities in Barcelona, but afterwards return to the community in Andorra.

In research, Andorra faces challenges typical of small countries, such as limited resources and the need to attract or maintain specialized talent. There is one campus-based public university (UdA) offering bachelor’s, master’s, and doctoral degrees, and several online private universities. An advanced health research centre at UdA is planned for early 2026. With its highly rated healthcare system, Andorra can serve as a testing ground for new medical technologies, telemedicine, and health data analytics. Besides some national registries, the country also has a Minimum Basic Data Set to which the national hospital is required to contribute. Andorra has robust data protection legislation (8).

As mentioned, Andorra has been part of WHO-EURO’s network of small countries since 2014 and was part of the 2016 Small Countries Health Information Network study. In the 8-country study (Andorra, Cyprus, Iceland, Luxembourg, Malta, Monaco, Montenegro, and San Marino), Andorra reported having available data in only 9 of the 21 health indicator categories, the least of the 9 countries and facing more small country challenges to health information systems – e.g., weak culture of integrating and using health information; lack of staffing and technical capacity; difficulties with data collection; and small numbers, large fluctuations, and wide confidence intervals – than the other countries. In short, while Andorra faces health data and research capacity challenges, it also has the inherent capacity to meet these challenges.

## METHODS

### The International Spinal Cord Injury survey (InSCI)

InSCI is the first global survey for persons with spinal cord injuries, initiated in 2017 by Swiss Paraplegic Research, Nottwil, Switzerland with the endorsement and support of the WHO. The data model and questionnaire structure were based on a Swiss SCI survey (SwiSCI), the development of which began in 2011. InSCI, like SwiSCI, is expressly built around the conceptual model of functioning found in the WHO’s *International Classification of Functioning, Disability and Health* (ICF) (9), and the ICF provides an international data reference framework for capturing all aspects and dimensions of the lived experience of a health condition, in this case SCI (10). InSCI is a joint effort of the International Spinal Cord Society (ISCoS) and the International Society of Physical and Rehabilitation Medicine (ISPRM) and has been designed to provide data for the implementation of the 2013 WHO report, *International Perspectives on Spinal Cord Injury* (IPSCI) (11). The study protocol, data model and other features of InSCI were published in a special issue of the *American Journal of Physical Medicine and Rehabilitation* (12).

The InSCI survey was first conducted in 2017–19 in 22 countries, representing all 6 WHO world regions. Selective results from comparative analyses were published in 2020 in the *Archives of Physical Medicine and Rehabilitation*. Beside a description of the cohort and sampling frames, these studies looked at environmental barriers for people with SCI in 22 countries (13), employment status (14), and quality of life (15). Later papers based on the 2017–19 InSCI looked at health inequality and income for people with SCI (16), the role of social relationships as a resource for mental health (17), and unmet health needs (18). This wide range of topics supported by the InSCI data reflects both the scope of the survey and the possibilities for extensive comparative research that the data from InSCI makes possible.

The second InSCI community survey, in which Andorra participated, was conducted in 2022–24 and a series of publications, covering an equally wide range of health topics, is in preparation, once again for *Archives of Physical Medicine and Rehabilitation*. As with the previous survey, the data from InSCI have been used by each country individually, and in comparisons between countries in the same region or countries that used a common additional module on a specific topic of interest (e.g., pain or exercise), to produce a growing body of international SCI literature. InSCI has proven to be a rich source of health-related data for the SCI research community, and also more generally for health researchers interested in comparative research on health systems and services issues for individuals with complex needs.

### The InSCI survey in Andorra

For the second InSCI survey, Andorra applied for inclusion in the international InSCI consortium, which has now grown to nearly 40 countries around the world. The Andorran government provided support, attracted in part by the fact that, with its small population, it might be possible to reach everyone in Andorra with SCI. An additional objective of the Minister of Health was to set in motion an initiative to develop a national SCI register. The Minister of Welfare also provided support to contact national patients’ associations for recruitment.

The sampling frame for the survey was constructed from an address list of all individuals with SCI and was compiled from different sources: patients were identified through hospital records, patient organization databases, requests for assistance to the Ministry of Welfare, and referrals from healthcare professionals such as physiotherapists, orthopaedic specialists, and general practitioners. In addition, the reference centres for SCI patients – Vall d’Hebron University Hospital in Barcelona and the Institut Guttmann (a neurorehabilitation centre) – also collaborated in identifying Andorran patients eligible to participate in the study.

Recruitment was based on the following inclusion and exclusion criteria: Eligible subjects are residents of the country, living with SCI in the community, and able to respond in the language(s) of the questionnaire. It was recommended that eligible persons be at least 12 months past medical and rehabilitation treatment and sustain long-lasting symptoms due to SCI. Excluded were those with spinal cord damage due to congenital aetiologies, such as spina bifida; or neurodegenerative disorders, such as multiple sclerosis (MS), or amyotrophic lateral sclerosis (ALS); or peripheral nerve damage, such as Guillain Barré Syndrome. Persons who are inpatients receiving first rehabilitation or first acute care at the time of the study are also excluded due to lack of community experience with SCI.

Andorra is an interesting example of how effective cohort recruitment can be because of geographic size and small population. Several strategies were used: a patient with SCI volunteered to help with contacting and recruiting other individuals with SCI. The patient was provided with a cell phone and an email was specially created for enabling contact with the InSCI team. A press conference was held in government facilities before the start to inform the general population. Formal meetings were also held with the Board of Physicians, nurses’ associations, and orthopaedic technicians. In the end, a total of 47 patients were identified. Given the comprehensive collaboration between the national health systems, welfare institutions, and reference centres, this likely represents all known SCI patients residing in Andorra. Nine of these passed away before the survey. One patient, because of a cognitive impairment due to dementia and unable to understand the questionnaire, was excluded; 3 did not consent to participate (although they did consent to be included in the National Register); all other participants did consent. Two individuals had moved to another country. In the end, this left a total of 32 participants. However, 1 of them considered herself to be totally recovered and was excluded by the InSCI study centre in Swiss Paraplegic Research that coordinates the InSCI study. In summary, the response rate was 32/35, that is, 91.4%.

## DISCUSSION

### Andorra as a living lab to address the challenges of small countries

Andorra’s participation in the second InSCI community survey has been a success. From the perspective of the overall study, the scope of InSCI has broadened and its epidemiological relevance for Europe enhanced as the Andorra data allow for analysis of the relative impact of geographic and population parameters on the experience of SCI. For Andorra itself, as future data analysis will disclose, researchers and policy-makers will have a clearer idea of the dynamics of the life experience of SCI in the community. Countries surrounding Andorra may learn key lessons concerning community-level services and support that, given the geographical proximity and the historical commonalities, may be attributable to sociopolitical, or economic, differences that can illuminate important policy differences. But the impact for Andorra in the mid- and long-term is also noteworthy, as participating in InSCI provides local researchers with invaluable experience in collecting and analysing data from an international cohort study, and creates interactions with researchers in other countries participating in joint publications and follow-up projects. Already, as noted, InSCI participation has motivated the government to begin the process of establishing a register for SCI, which will strengthen Andorra’s SCI knowledge base and provide evidence for reforms in treatment and policy.

## CONCLUSION

Andorra’s participation in InSCI as a multi-country community cohort study has been a success for all parties. Without compromising methodological standards, Andorra has contributed to a powerful SCI database that can be used for studies covering a wide range of topics, across countries, and in some instances longitudinally as well. WHO EURO’s initiatives concerning small countries, especially the ongoing SCHIN project and its focus on developing data streams for key health indicators, can benefit from the example of Andorra participating, with other larger countries in the region, in InSCI. In this sense, Andorra’s participation is a natural experiment exploring one way in which the data and research capacity problem of small countries can be addressed. Moreover, given its compact geography and population, Andorra can be an ideal “living lab” for testing out implementation of innovative approaches to community-based services for persons with SCI. By embracing its role as a living lab, Andorra is not only advancing research but also positioning itself as a global leader in sustainable innovation and development.
